# Evaluation of Sequential Processing for the Extraction of Starch, Lipids, and Proteins From Wheat Bran

**DOI:** 10.3389/fbioe.2019.00413

**Published:** 2019-12-13

**Authors:** Roya R. R. Sardari, Samuel Sutiono, Hafiz Abdul Azeem, Mats Galbe, Mats Larsson, Charlotta Turner, Eva Nordberg Karlsson

**Affiliations:** ^1^Division of Biotechnology, Department of Chemistry, Lund University, Lund, Sweden; ^2^Chair of Chemistry of Biogenic Resources, Technical University of Munich, Munich, Germany; ^3^Centre for Analysis and Synthesis, Department of Chemistry, Lund University, Lund, Sweden; ^4^Department of Chemical Engineering, Lund University, Lund, Sweden; ^5^Lantmännen R&D, Stockholm, Sweden

**Keywords:** wheat bran, bran refinery, fractionation, food enrichment, extraction, sequential processing

## Abstract

In line with the need to better utilize agricultural resources, and valorize underutilized fractions, we have developed protocols to increase the use of wheat bran, to improve utilization of this resource to additional products. Here, we report sequential processing for extraction of starch, lipids, and proteins from wheat brans with two different particle sizes leaving a rest-material enriched in dietary fiber. Mild water-based extraction of starch resulted in maximum 81.7 ± 0.67% yield. Supercritical fluid extraction of lipids by CO_2_ resulted in 55.2 ± 2.4% yield. This was lower than the corresponding yield using Soxhlet extraction, which was used as a reference method, but allowed a continued extraction sequence without denaturation of the proteins remaining in the raw-material. Alkaline extraction of non-degraded proteins resulted in a yield corresponding to one third of the total protein in the material, which was improved to reach 62 ± 8% by a combination of wheat bran enzymes activation followed by Osborne fractionation. The remaining proteins were extracted in degraded form, resulting in maximum 91.6 ± 1.6% yield of the total proteins content. The remaining material in both fine and coarse bran had a fiber content that on average corresponded to 73 ± 3%. The current work allows separation of several compounds, which is enabling valorization of the bran raw-material into several products.

## Introduction

Wheat bran is a by-product from the milling process in the production of refined grains and has an estimated annual production of ~100–150 million tons/year world-wide (Hell et al., 2014). Bran makes up the outer layer of wheat grains and consists of the tissues termed aleurone, hyaline, testa, and pericarp (Prinsen et al., 2014). The main components of wheat bran tissues include starch (15–25%) and non-starch polysaccharides including dietary fibers (50–60% of which 52–70% is arabinoxylan, 20–24% is cellulose and ~6% is ß-glucan), lignin (6–12%), proteins (10–25%), lipids (2–4%), and minerals (3–8%) (Apprich et al., 2014; Hell et al., 2014). The composition is prone to variation that depends on a combination of natural fluctuations, extraction, and analytical methods, and also on which tissue of the bran that is mainly released. The outer bran layer, the pericarp, is composed of mainly insoluble dietary fibers and lignin. Lignin is also present in the seed coat (testa and hyaline) which also contains the antioxidizing alkylresorcinols, while the aleurone layer (the inner layer next to the starchy endosperm) contains a mixture of proteins, fibers, lipids, and minerals (Javed et al., 2012; Onipe et al., 2015). Due to the large quantities of bran produced, this material has potential for production of higher value products (Sozer et al., 2017). A number of refined molecules have been suggested as potential products from a bran refinery, and are normally divided according to the main bran component (Apprich et al., 2014; Pruckler et al., 2014).

Currently, the main use of bran is as low-value ingredient for human and animal consumption (Prinsen et al., 2014; Pruckler et al., 2014). The comparatively lower use as an ingredient in food is related to sensory attributes and texture, for instance a bitter taste related to the presence of phenolic compounds, limited shelf-life due to lipids turning rancid upon oxidation and incompatibility with certain food matrices. It is clear that a number of potentially interesting products can be made from wheat bran, but to allow increased utilization, some of these compounds need to be separated and unfavorable tastes need to be removed.

Starch is one of the main components in wheat bran, and is found in two different types of granules. The chemical composition and functional properties of the wheat bran granules have been reported to differ from granules of commercial wheat starch, which gives starch from wheat bran unique properties (Xie et al., 2008; Liu and Ng, 2015). In addition, lipids from wheat bran are reported to be of potentially high value for food and pharmaceutical industries due to having essential unsaturated fatty acids (Jung et al., 2010; El-Shami et al., 2011; Lei et al., 2018). Extraction of lipids from wheat bran is also needed to avoid rancidity in other fractions, since these compounds have a tendency for oxidation and degradation (Merali et al., 2015). Wheat bran, with 13–18% of proteins, is a potentially valuable source for food industry and can be used as an additive in a variety of food products such as beverages, meat, etc. (Sozer et al., 2017). Today, the interest in replacing the expensive and limited dietary proteins with a cheap source of protein from plants is increasing due to the increasing world population (Apprich et al., 2014).

Although the literature holds a significant number of analytical fractionation methodologies, most of the experimental studies have had a focus on obtaining a single fraction, often favoring the carbohydrate fraction (Hell et al., 2014; Jefferson and Adolphus, 2019), which is the largest part of the bran. A range of methods can be used to obtain each fraction (with differences in how harsh the treatment is). Recently, some reviews have considered a biorefining approach using wheat bran as raw material (Soukoulis and Aprea, 2012; Reisinger et al., 2013, 2014; Celiktas et al., 2014; Tirpanalan et al., 2014), but their focus was on obtaining monosaccharides as products using different types of (combined) technologies (e.g., organosolv, acid hydrolysis, hydrothermal, or enzymatic degradation processes). and monitoring the effects of processing on the overall composition of the remaining bran product mixture (Reisinger et al., 2013; Pruckler et al., 2014).

The aim of this study is to find and elaborate suitable technologies for sequential processing which have the ability of fractionating a component without too much losses or destruction of other components. Therefore, different methods were modified, combined and tested for extraction of wheat brans components and also the highest possible extraction yields of the components were evaluated.

## Materials and Methods

### Materials

Reagents and chemicals were supplied by Sigma-Aldrich (Sweden) unless otherwise stated.

Two types of milled wheat bran; fine bran with a particle size of < 1 mm and coarse bran with an average particle size of 1.5 mm were obtained from Lantmännen Reppe AB (Lidköping, Sweden). Carbon dioxide (99.9993%) with a dip tube was purchased from Linde, Sweden.

**Scheme 1:**
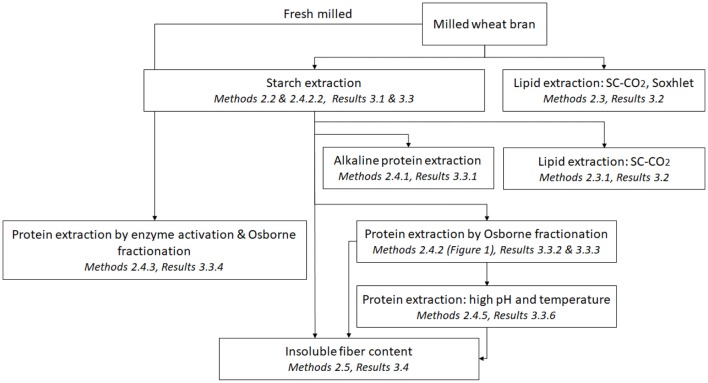
Flowchart overviewing the different sequential processes used in this study.

The sequential processing alternatives are shown in Scheme 1, and described in the methods part. The experiments (unless otherwise stated) were performed using three independent replicates and reported as mean ± standard deviation.

### Extraction of Starch From Wheat Bran

One hundred milliliters tap water with a temperature of 30°C was added to a 250 mL baffled shake flask containing a 10 g sample of fine or coarse bran, respectively. The flasks were incubated in a shaker incubator (Infors HT, Ecotron) at 30°C for 30 min with constant shaking. The water fraction and the wheat bran residues were collected using vacuum filtering. The bran residues were then washed with 500 mL tap water, and dried in a 50°C oven overnight.

The same experiment was then repeated using tap water with a temperature of 55°C and an incubation time of 4 h. The starch content in the bran residues collected from both experiments was analyzed before and after extraction.

### Extraction of Lipids From Wheat Bran

#### Supercritical Carbon Dioxide Extractions

Supercritical fluid extraction (SFE) was performed using supercritical carbon dioxide (SC-CO_2_) as solvent. A laboratory scale SC-CO_2_ extraction system was used as described by Turner et al. (2001). A wheat bran sample (1 g) was placed in a stainless-steel extraction vessel. The lipid extraction was studied at 50°C at a pressure of 150 and 350 bars, respectively. The density of SC-CO_2_ at 50°C was 0.7 g/mL at 150 bars, and 0.9 g/mL at 350 bars. The CO_2_ flow rate was 1 mL/min during the extraction period of 2.5 h and the defatting rate was followed by collecting the extracted oil every 30 min during the entire extraction period. At the end of the extraction, 10 mL of acetone was used to flush the system along with CO_2_ with a flow rate of 2 mL/min for 15 min. Then, acetone was evaporated completely from the collection vessel under a nitrogen evaporator. Collected oil samples and wheat bran residues were kept for further analysis. A *t*-test for independent means was made to analyse the significance of yield differences.

Two lipid extraction experiments were also carried out using SFE on de-starched fine and coarse wheat brans, respectively. These were set-up as single trials, and lipid extraction was performed at 50°C, 150 bars for 2.5 h. Collected oil samples and wheat bran residues were kept for further analysis.

#### Soxhlet Extraction

Soxhlet extraction was performed as a reference method (Association of Official Analytical Chemists, 1995; Shahidi, 2003) on wheat bran samples (5 g) at 80°C (refer to ~150 drops/min) for 8 h using 150 mL *n*-hexane. Then, *n*-hexane was removed from the samples using a rotary vacuum evaporator (Heidolph). The extracts were stored at −20°C for further analysis.

### Extraction of Proteins From Wheat Bran

#### Mild Alkali Extraction of Wheat Bran Proteins

Extraction of proteins, from fine and coarse brans, was carried out at 25°C after extraction of starch. In a screening procedure, four samples of fine and course brans (5 g portions) were put into eight 250-mL baffled shake flasks. After de-starching, as described in section Extraction of Starch From Wheat Bran, the wet bran samples were subjected to protein extraction. For protein extraction 50 mL buffer (at pH 8 using 50 mM sodium phosphate buffer, and at pH 9 and 10 using 50 mM borate buffer) was added to the respective sample, followed by incubation at four residence times (1, 6, 12, and 24 h), in a shaker incubator (Infors HT, Ecotron) at 25°C, 150 rpm. At each time point, two flasks (fine and coarse bran sample) were removed from the incubator, the liquid phase was separated from the bran residue (solid phase) using vacuum filtration, and the bran residue was washed with 500 mL of tap water using vacuum filtration. The bran residue was finally dried at 50°C overnight for analysis of protein content.

#### Osborne Fractionation

**Figure 1:**
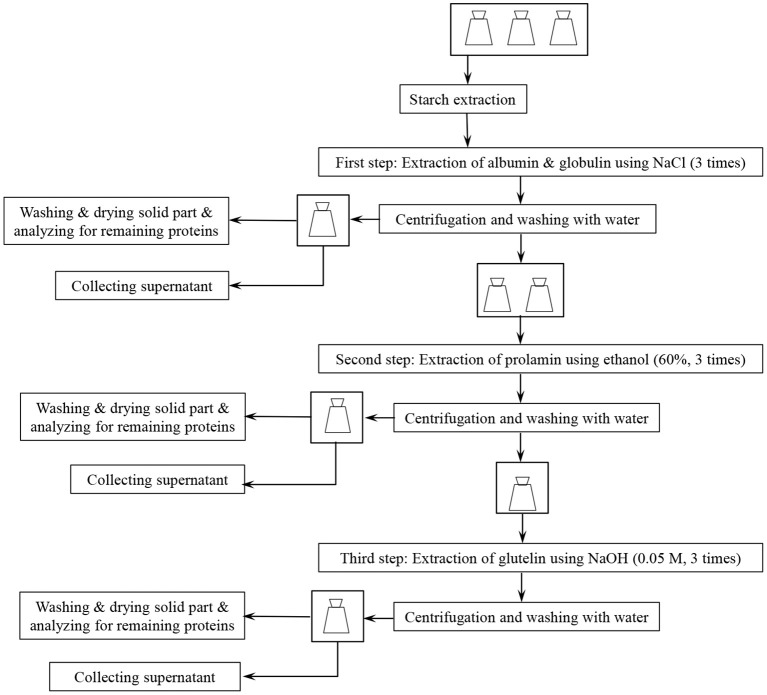
Flowchart for Osborne fractionation method for extraction of wheat bran proteins.

Figure 1 presents a flow chart for Osborne fractionation. A separate starch extraction step (Figure 1) was initially applied in the fractionation sequence (see section Osborne Fractionation of De-starched Wheat Brans Proteins), but starch separation was later combined with the first protein fractionation step (using 0.4 M NaCl, see section Co-extraction of Wheat Brans Starch and Proteins by Osborne Fractionation).

##### Osborne fractionation of de-starched wheat brans proteins

Fine and coarse wheat brans samples (2.5 g) were first de-starched (section Extraction of Starch From Wheat Bran) in six 250-mL baffled flasks. Then the bran residues were transferred to six 50-mL falcon tubes and suspended in 25 mL 0.4 M NaCl in 50 mM sodium phosphate buffer, pH 7.6. The tubes were placed horizontally in a shaker incubator (Infors HT, Ecotron) at 25°C, 200 rpm for 20 min followed by centrifugation (3,890 × g, 10 min in a Sigma 3-16PK centrifuge). The extraction was repeated three times, and the supernatants were collected for protein analysis. Then the bran residue was mixed with 30 mL distilled water, and centrifuged (3,890 × g, 10 min, Sigma 3-16PK). The solid residues from one coarse bran sample and one fine bran sample were then collected, washed with 500 mL of distilled water using vacuum filtration and dried at 50°C overnight and saved for analysis of proteins after the first step.

The second extraction step (Figure 1) was done by adding 25 mL 60% (V/V) ethanol to the remaining four samples followed by shaking at 25°C, 20 min and centrifugation (as above). The incubation was repeated three times and the supernatants were collected for protein analysis. Subsequently, 30 mL distilled water was added to the bran residues, followed by mixing, and centrifugation (3,890 × g, 10 min, Sigma 3-16PK). The residues of one coarse bran sample and one fine bran sample were again collected, washed with distilled water and dried (as described for step one above) for analysis of proteins after the second step.

Finally, 25 mL of warm (60°C) 0.05 M NaOH was added to the two remaining samples followed by shaking at 60°C for 20 min and centrifugation (as above). This was repeated three times and the supernatants were collected for protein analysis. The remaining residues were washed as above with distilled water, dried, and saved for protein analysis.

##### Co-extraction of wheat brans starch and proteins by Osborne fractionation

The Osborne fractionation described in section Osborne Fractionation of De-starched Wheat Brans Proteins was also carried out for both fine and coarse wheat brans without the separate de-starching step (Figure 1). In this case, the procedure was carried out at 25°C and the starch and protein extracted in the supernatant in step one were separated by filtration, followed by analysis of starch and total protein. After each of the three extraction steps (performed as described above), the residues were washed with 500 mL of distilled water using vacuum filtration and dried at 50°C overnight and saved for protein analysis.

#### Combination of Wheat Bran Enzymes Activation and Osborne Fractionation

This experiment was performed using fresh milled fine wheat bran. Two temperatures (30 and 50°C) and two pH (4.5 and 7) and four solid-liquid ratios (1:1, 1:2, 1:3, 1:4) were chosen for investigation of the endogenous enzymes activity. First, organic acid solutions containing lactic acid and acetic acid at a 4:1 molar ratio, at two different pHs (4.5 and 7) were prepared and divided into different volumes (2.5, 5, 7.5, and 10 mL) in 50-mL falcon tubes. Then, 2.5 g of fresh milled fine wheat bran was added to each falcon tube. The samples were mixed and incubated (Infors HT, Ecotron) at two different temperatures (30 and 50°C) for 24 h. Then Osborne fractionation (three steps, Figure 1) was applied immediately to all samples which were analyzed for remaining protein. The screening was first made using single sample sequences, and the condition showing highest amount of extracted protein was then repeated twice.

#### Precipitation of Extracted Protein From the Osborne Fractionation Collected Supernatants

The pH of all collected supernatants from the Osborne fractionation was adjusted to 4.5 using 3 M HCl and then the suspensions were centrifuged (3,890 × g, 10 min, Sigma 3-16PK) to separate precipitates from the liquid. The precipitates were washed with distilled water, centrifuged to remove water, and freeze dried for further analysis.

#### Extraction of Proteins at High pH and High Temperature

The solid wheat bran residues (2.5 g) obtained after step 3 in Osborne fractionation (Figure 1) were added to 25 mL of 0.05 M sodium hydroxide and the suspension was mixed and heated to 120°C on a magnetic hot plate (Heidolph) for 1 h with constant stirring using a magnet. Then the residues were washed with 500 mL of distilled water using vacuum filtration and dried at 50°C overnight and analyzed for protein by the Dumas method (section Protein Analysis, below).

### Quantitative Analyses

#### Starch Analysis

Starch determination in wheat bran was performed using a total starch assay kit (Megazyme) based on the use of two enzymes: thermostable α-amylase and amyloglucosidase. Briefly, the starch was hydrolyzed to soluble branched and unbranched maltodextrin using thermostable α-amylase at pH 5, 100°C, 6 min, using a heating block (Techne Dri-block). Then maltodextrin was hydrolyzed to *D*-glucose using amyloglucosidase at 50°C, 30 min in the heating block. Finally, the glucose was oxidized to *D*-gluconate and the released hydrogen peroxide was converted to quinone-imine dye using peroxidase (GOPOD reagent enzymes) and then the absorbance of the dye was measured at 510 nm by a UV/ Visible spectrophotometer (Ultrospec 1000, Pharmacia biotech) with *D*-glucose as standard (total starch assay procedure, Megazyme, Ireland).

#### Analysis of Total Lipids

Total lipid analysis was done by liquid-liquid extraction using a mixture of chloroform, methanol, and water as described by Bahrami et al. (2014). Six milliliters of chloroform-methanol solution (1:2 v/v) and 1.5 mL of distilled water were added to 0.25 g wheat bran sample in a 15-mL falcon tube and the suspension was mixed for 2 min. Then 2 mL of water and 2 mL of chloroform were added to the mixture. The mixture was shaken vigorously and then centrifuged for 10 min at 3,890 × g. The bottom phase (organic layer) was transferred into a new 15-mL pre-weighed falcon tube. The 6 mL of chloroform and 0.2 mL of acetic acid were added to the water phase, mixed vigorously, and centrifuged; then the bottom layer was added to the first organic layer. The chloroform was evaporated, the falcon tube containing sample was weighed and the total lipids content was determined by gravimetric analysis.

#### Fatty Acid (FA) Analysis

Determination of FA compositions was performed by methylation according to the method described by Svensson and Adlercreutz (2011). Approximately 5 mg of fatty acids were weighed and dissolved in 1 mL cyclohexane and was then was added to 500 μL 0.5 M sodium methoxide and incubated in a heating block (Techne Dri-block) at 50°C, 30 min. The reaction was stopped by adding 2 mL water saturated with sodium chloride. After vortex mixing and centrifugation, the upper layer was transferred to a chromatography vial for analysis. For gas chromatography (GC) analysis a silica column (Supelco, 60 m × 0.25 mm × 0.02 μm) was used. Initial column temperature was 160°C with the heating rate of 3°C/min until 250°C. Total analysis time was 40 min and the column pressure was 20.00 psi. A FID detector was used with the temperature of 270°C. The helium, hydrogen, and air flow rate were 25, 30, and 300 mL/min, respectively.

#### Protein Analysis

Total protein determination was performed using the Kjeldahl (Jones, 1991) and Dumas (Saint-Denis and Goupy, 2004) methods. In the Kjeldahl method, the sample was digested (Tecator 2006 digestor) using concentrated sulfuric acid. Then, the ammonia gas was released to a solution containing diluted sulfuric acid using a distillation system (Tecator 1002 distilling system) and the obtained solution was titrated by diluted NaOH solution to get the nitrogen content. In the Dumas method; using an elemental analyser (Flash EA 1112, Thermo Fisher Scientific, USA), combustion of samples was done at high temperature (over 1,000°C) in presence of pure oxygen and the quantitative conversion of obtained nitrogen oxides to N_2_ was performed via a reduction chamber containing copper heated to around 650°C. Other volatile combustion products (water and CO_2_) were either trapped or separated. Finally, the nitrogen gas was measured using a thermal conductivity detector. The nitrogen content was multiplied by 6.25 (empirical protein factor) to get the protein content in the wheat bran (AACC 46-11A). All the proteins measurements were from solid parts and the proteins yields were weight of extracted proteins per weight of total initial proteins.

The molecular weights of the native extracted proteins in the supernatant was determined by size exclusion chromatography (SE-HPLC) (Hancock, 1984; De Brier et al., 2015) using a Dionex HPLC system (Ultimate-3000 RSLC, Dionex) with a column (Yarra 3u SEC-2000, 300 × 4.6 mm, Phenomenex) and 50 mM sodium phosphate buffer at pH 6.8 as mobile phase. The flow rate was 0.5 mL/min and the column oven was maintained at 25°C. Eluted proteins were detected at 280 nm using a UV-Vis detector (Ultimate 3000 RS, Dionex). Aprotinin, alcohol dehydrogenase, carbonic anhydrase, and albumin (Gel filtration marker kit, Sigma) were used as standards.

The molecular weight of the denatured extracted proteins was determined using sodium dodecyl sulfate-polyacrylamide gel electrophoresis (SDS-PAGE) as described by Laemmli (1970) using pre-casted gels (Bio-Rad, USA). Twenty microliters of sample from the supernatant was added to 5 μL of Laemmli sample buffer. The sample buffer for the supernatant from the third step of the Osborne fractionation contains DTT (1,4-dithiothreitol, 1% w/v) as a reducing agent. All samples were mixed, heated at 100°C for 10 min, and centrifuged at 9,600 × g for 2 min (Eppendorf microcentrifuge 5424). To each well, 15 μL of supernatants were loaded and subjected to electrophoresis at a current of 30 mA for around 45 min. A protein molecular weight marker (10–250 kDa protein standards, Bio-Rad) was loaded into one of the wells. After separation, the gel was placed in staining solution for 30 min. The staining solution consisted of 1 g of Coomassie 0.2%, 200 mL of MeOH 40%, 50 mL of HAc 10%, and 260 mL of milliQ-water. Then the gel was de-stained using de-staining solution (40% methanol, 10% acetic acid, and 50% water) and molecular weight of the samples were determined using the SDS-PAGE marker (Biorad).

Fourier transformed infrared (FT-IR) spectroscopy was used to determine the functional groups of the protein precipitates from the supernatants. Infrared spectra of the protein precipitates fractions were recorded in the 4,000–400 cm^−1^ region using a FT-IR system (Nicolet is5, Thermo Fisher Scientific).

#### Insoluble Fiber Analysis

The insoluble fiber determination was done using a 3-step enzymatic reaction (Megazyme K-TDFR 10/14). In the first step, the sample was treated with heat stable α-amylase at 100°C, 30 min with constant shaking in a thermoshaker (HLC, Ditabis) for gelatinization, hydrolysis, and depolymerization of starch. Then, after cooling to 60°C, protease was added, and the samples were again incubated in the thermoshaker (HLC, Ditabis) at 60°C with constant shaking to solubilize and depolymerize proteins. In the next step, amyloglucosidase was added the incubation was continued at 60°C, 30 min to hydrolyze starch fragments to glucose. Thereafter, the residue was filtered, washed with hot water (70°C), ethanol (95%), and acetone, respectively and dried in an oven at 100°C and weighed.

## Results

Hammer milled wheat bran fractions of two particle sizes were used as starting materials, and processing methodologies to enrich starch, lipids, and proteins were chosen, prioritizing mild technologies, to allow non-destructive processing in several steps (Scheme 1).

### Extraction of Starch From Wheat Bran

**Figure 2:**
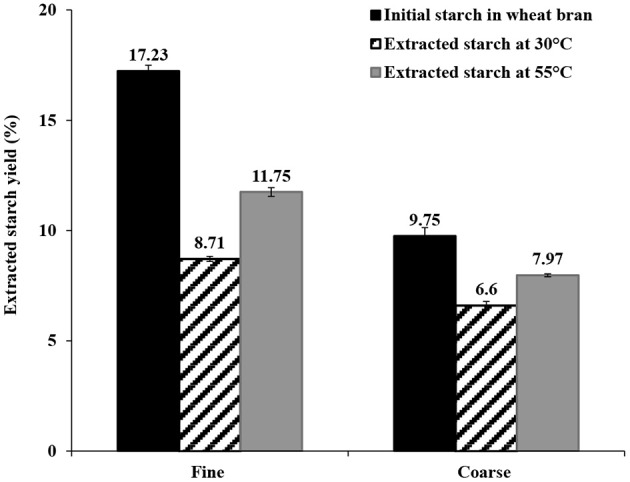
The extracted starch yields from fine and coarse wheat brans by swelling in water at 30°C for 30 min and 55°C for 4 h. The data in the figure is given as an average ± std deviation of three experiments and shown as % starch (dry weight) of bran dry weight.

Extraction of starch from wheat bran using water as solvent was evaluated at two different temperatures and times (30°C for 30 min and 55°C for 4 h). The extractions at 30°C for 30 min and 55°C for 4 h resulted in isolation of 50.6 ± 0.65 and 68.2 ± 1.2% of the starch in fine bran and 67.7 ± 1.9% and 81.7 ± 0.67% of the starch in course bran, respectively (Figure 2). The major part of the extracted starch in the water phase at 30°C was dispersed in insoluble form (20 μm ≤ extracted starch ≤ 100 μm) from both bran sizes (fine and coarse), allowing separation from the remaining bran (solid part) by filtration. The filtered starch was then sedimented or centrifuged from the water phase as an insoluble starch fraction. Only a small amount of starch that was solubilized in the water phase at 30°C (5 and 6% of the total starch from fine and coarse brans, respectively).

Extraction at 55°C resulted in significantly higher solubilization in water (27 and 25%, from fine and coarse brans, respectively), and was less favorable from a separation perspective (more difficult to remove water and difficult to separate starch from protein, as explained in section Extraction of Proteins From Wheat Bran below).

The yield of extracted starch from coarse bran was surprisingly higher than that from fine bran in both extraction methods, despite the larger particle size. Starch extraction by water is a mild non-destructive extraction method, advantageous to apply at an early stage in a processing cascade, and is here suggested as a first step in the processing chain (Figure 1).

### Extraction of Lipids From Wheat Bran

**Figure 3:**
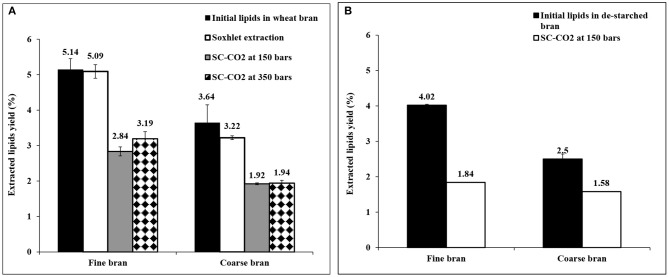
Extracted lipids from fine and coarse wheat brans **(A)** using SC-CO_2_ and Soxhlet extraction methods without extraction of starch, **(B)** using SC-CO_2_ extraction method after extraction of starch. The total lipids were determined using the chloroform-methanol-water extraction method, as indicated in section Analysis of Total Lipids, and targets a broader range of lipid compounds than the used extraction methods due to extracting more polar lipids which naturally gives lower yield when SC-CO_2_ or Soxhlet are used. The figure shows the average ± std deviation of three experiments (except for SC-CO_2_ extracted lipids from de-starched bran in **(B)**, which was a single experiment), the data is given as % lipids (dry weight) of bran dry weight.

Extraction of lipids from bran of the two particle sizes (without prior starch extraction) was carried out at two different pressures in order to investigate the optimum pressure for extraction of lipids with high yield. As seen in Figure 3A, the total yield of extracted lipids at 150 bars and 350 bars were 55.2 ± 2.4 and 62.1 ± 3.9% from fine bran and 52.8 ± 0.73 and 53.3 ± 2.2% from coarse bran, respectively. The yield of extracted lipids did not increase with the increase in SC-CO_2_ pressure using course bran and the increase in extraction yield for fine bran was not statistically significant (*P* < 0.05). Thus, the condition at 150 bars was considered most favorable for continued trials.

Soxhlet extraction in hexane, was also applied as a reference method (Association of Official Analytical Chemists, 1995), and was more effective, resulting in higher yield of extracted lipids than SC-CO_2_ extraction (Figure 3A). In this case co-extraction of starch was shown together with the lipids (data not shown), highlighting the advantage of extracting the starch as a first step in the extraction sequence.

The lower pressure (150 bars) was then selected for experiments, using de-starched bran (from section Extraction of Starch From Wheat Bran). Composition analysis however showed that some lipids were lost, corresponding to 21.8 ± 0.55% of the lipids in fine bran and 31.3 ± 3.9% of the coarse bran lipids (Figure 3B). The resulting lipid extraction yield from de-starched fine bran (46%, Figure 3B) was hence lower than the yield from untreated wheat bran (55%, Figure 3A), and some lipids are judged to be co-extracted with the starch, despite using water as solvent. In coarse bran, the trend was opposite, and the yield of extracted lipids after de-starching was higher (63% of the lipid content after de-starching, Figure 3B) than the yield obtained without de-starching (52%, Figure 3A) indicating that the bran particles were affected by the de-starching treatment.

**Figure 4:**
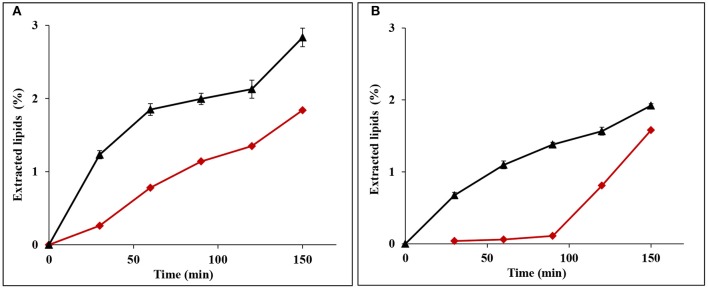
Profile of defatting rate of **(A)** fine and **(B)** coarse wheat brans during lipid extraction by SC-CO_2_. Symbols indicate (▴) wheat brans without de-starching (average ± std deviaton of three experiments) and (♦) wheat brans after de-starching (single experiments). The data is given as % of bran dryweight.

The above statement is strengthened by the lipids extraction behavior from the bran, which was dependent on the extraction sequence. Without prior starch extraction, the rate of defatting was high during the first hour, and then decreased slowly, in both fine and coarse bran. In contrast, lipid extraction rate in de-starched fine bran was lower, but continued to increase during the second hour and adopted an almost a linear rate during the run time. In de-starched coarse bran, the lipid extraction started after 1 h and then increased with a linear rate. Since the defatting increased significantly toward the end of the run time in both de-starched fine and coarse brans, a longer lipid extraction procedure after de-starching is necessary (Figure 4).

Fatty acid (FA) profiling by gas chromatography showed that linoleic acid (C18:2) was the most abundant fatty acid in the extracts and constituted around 60% of the total fatty acids. Palmitic acid (C16), oleic acid (C18:1), and α-linolenic acid (C18:3) were the second, third, and fourth most abundant FAs in the extracts, and corresponded to 16, 14, and 6% of the total FAs, respectively, which is in agreement with the results described by Cardenia et al. (2018). There was no difference in the content of linoleic acid regardless of the method used. Palmitic acid content was lowest when Soxhlet extraction of coarse bran was used. Extraction of lipids from fine bran using SC-CO_2_ with the pressure of 150 bars gave the highest amount of oleic acid, but the lowest α-linolenic acid content (Supplementary Table 1).

### Extraction of Proteins From Wheat Bran

The protein-shift (a transition in the protein consumption balance with less animal proteins and more plant-based proteins) highlights the need of efficient utilization of proteins from agricultural resources. Proteins can be extracted in either degraded or non-degraded forms, the latter with a higher value. Thus, different methods were employed to maximize the extraction of protein from bran.

#### Alkaline Extraction of Proteins

**Figure 5:**
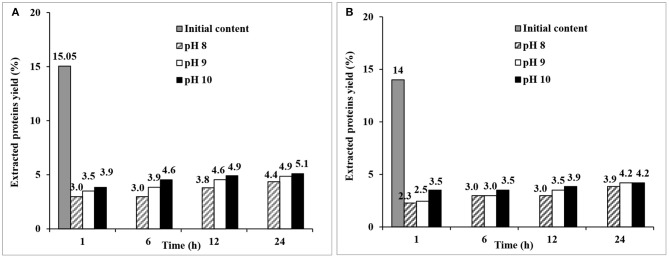
Protein extraction yield from **(A)** fine and **(B)** coarse de-starched wheat bran at 25°C, at three different pHs (8, 9, 10) and at four residence times (1, 6, 12, and 24 h). The figure shows single experiments.

Alkaline extraction of proteins on de-starched material was made at relatively mild conditions (25°C, not exceeding pH 10 to prevent degradation of native proteins), and showed a trend of increasing protein extraction yield with increasing pH and time for both fine and coarse brans (Figure 5). The highest yields in the extraction series (34 and 30% of the protein content for fine and coarse bran, respectively) were obtained at at the highest pH (Figure 5). The protein extraction yield from fine bran was higher than that from coarse bran at the corresponding pH and time. These results showed the effect of particle size, where the smaller size with higher surface to volume ratio seemed beneficial.

#### Osborne Fractionation

To improve the yield of extracted proteins, a modified Osborne fractionation procedure (Figure 1) was performed on both fine and coarse brans, involving de-starching combined with three extraction steps (0.4 M NaCl, 60% ethanol, and 0.05 M NaOH) to promote extraction of proteins with differing solubility.

**Figure 6:**
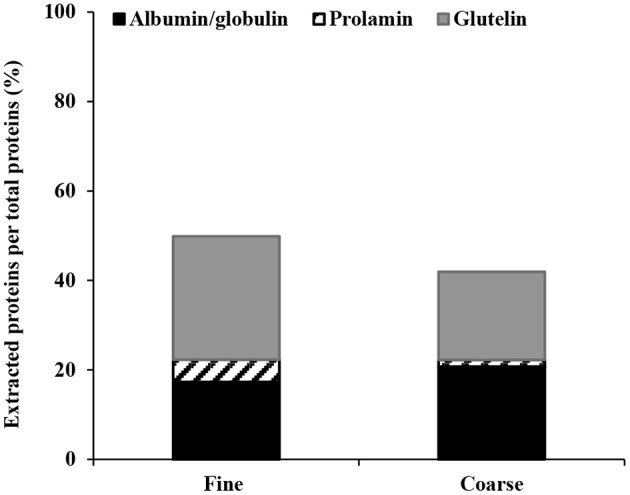
Proteins extraction yield from fine and coarse wheat brans, after using Osborne fractionation method. The average of three experiments are shown for each fraction.

The total extraction yield of fine bran proteins increased to 50 ± 1.8% (Figure 6), and the glutelin fraction (obtained after step three) corresponded to the largest protein proportion (36% of the proteins remaining from the second step, and 27.9% of the total proteins). This was followed by the water soluble albumin/globulin fraction (totally 17.2%), of which 9.6% was extracted into the water-phase during de-starching, and an additional 8.5% of the protein remaining after starch extraction (7.6% of the total protein) was extracted in the first Osborne fractionation step. The prolamin fraction obtained after extraction step two was smallest and corresponded to 6.1% of the protein remaining after the first step (5.1% of the total protein; Figure 6).

The total yield of extracted proteins from this sequence using coarse bran was 42 ± 0.005% (Figure 6). The water-soluble albumin/globulin (20.7% of the total protein) was extracted both during the de-starching step (12.5% of the total proteins) and the first Osborne fractionation step (9.4% of the remaining protein from starch extraction step, corresponding to 8.2% of the total proteins). The prolamin fraction from the second extraction step corresponded to 1.9% of the remaining proteins from the first step (1.5% of the total proteins). The glutelin extraction yield from step three corresponded to 25.3% of the remaining protein from step two (19.6% of the total protein). The total protein extraction yield from fine bran was 8% higher than from coarse bran, which shows the influence of the milling on the extraction yield.

#### Co-extraction of Starch and Proteins by Osborne Fractionation

Simultaneous extraction of wheat bran starch and proteins was performed by removing the separate de-starching step and use the first extraction step in the Osborne fractionation sequence both for extraction of water/salt-soluble proteins and for removal of starch (by sedimentation). The fine and coarse wheat brans were in the modified scheme first extracted with salt-containing buffer (instead of water) at pH 7.6, and the results showed protein extraction yields of 48.8 ± 0.05 and 41.9 ± 0.4% for fine and coarse brans, respectively, which were comparable with the protein extraction yields from de-starched fine (50 ± 1.8%) and coarse brans (42 ± 0.005; section Osborne Fractionation). The starch extraction yields in the co-extraction process was 50.6 ± 2.3 and 60.8 ± 4.1% for fine and coarse brans, respectively, which was similar to the 50.6 ± 0.65% yield from fine bran, and only slightly lower than that obtained from coarse bran (67.7 ± 1.9%) if a separate starch extraction step at 30°C for 30 min was employed (section Extraction of Starch From Wheat Bran). The difference observed for course bran may depend on batch variations, due to minor variations in temperature and pH (as the previous de-starching was without buffering), proving that the sequence can be shortened without loss.

#### Combination of Wheat Bran Enzymes Activation and Osborne Fractionation to Increase Proteins Extraction

**Figure 7:**
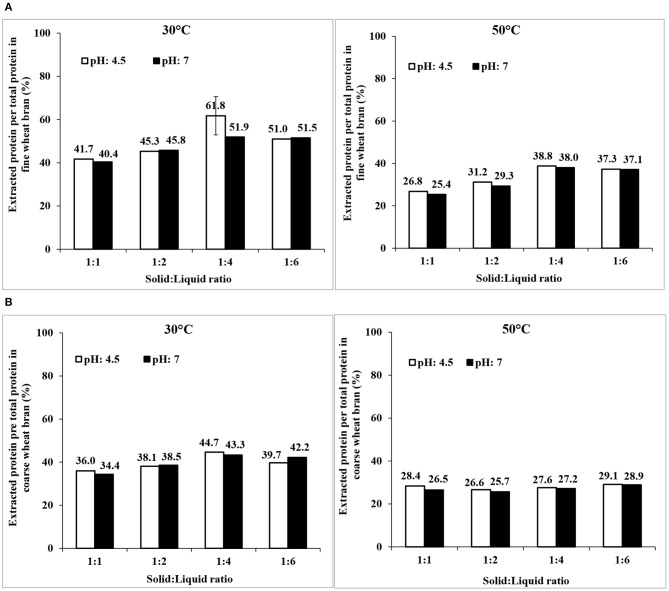
Extraction of wheat bran proteins by activation of wheat bran endogenous enzymes in **(A)** fine and **(B)** coarse wheat brans followed by Osborne fractionation. The data shown are results of single trials, that were repeated twice at the condition showing highest extraction yield (fine bran, 30°C, pH4.5, 1:4 solid liquid ratio), and there shown as mean ± std deviation of three trials.

The possibility to increase protein extraction yields by activation of endogenous wheat bran enzymes (by incubation at different temperatures, pH, and solid:liquid ratios) prior to the Osborne fractionation procedure was screened. It should be noted that fresh milled brans were necessary, and were used in order to have active enzymes. As seen in Figure 7, the maximum yield of extracted proteins was increased to 62 ± 8% for fine bran (at the best condition 30°C, pH 4.5, and a solid: liquid ratio of 1:4) but remained relatively constant for coarse bran (estimated to 45% from a single trial) compared to using Osborne fractionation without enzyme activation. Thus, activation of enzymes increased the extraction yield of non-degraded protein by 12% in fine bran, but only a few percentages in coarse bran. The drawback of this methodology is the need of using freshly milled bran, in order to achieve the desired activation. Use of milled bran, stored for a longer period (>1 month) did not result in the desired activation of endogenous enzymes (data not shown).

#### Characterization of Extracted Proteins Obtained From Osborne Fractionation

**Figure 8:**
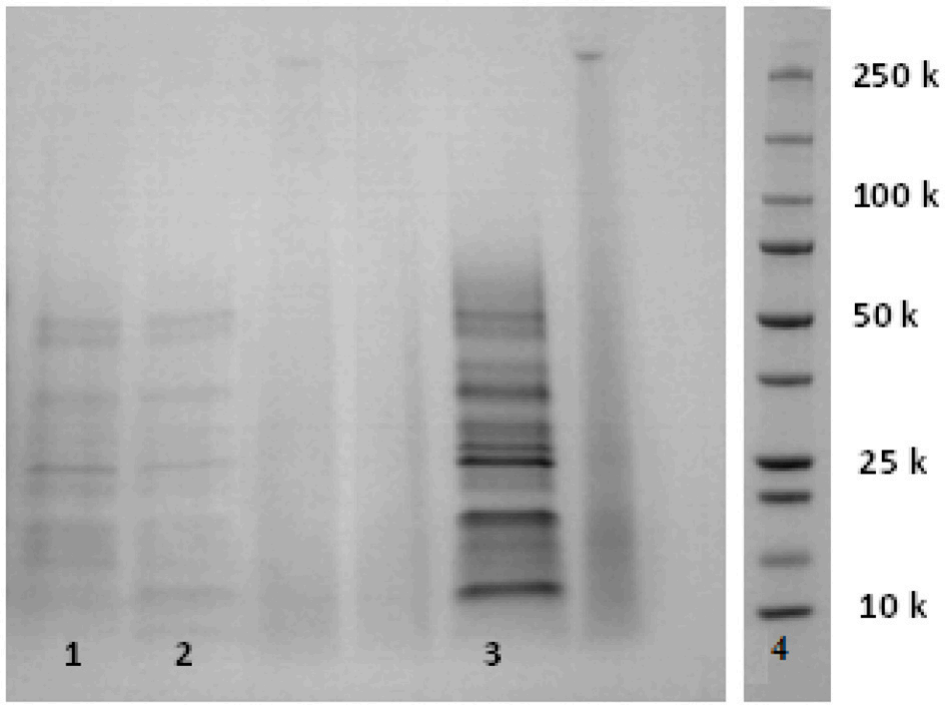
SDS-PAGE profile of albumins/ globulins in (1) fine bran, (2) coarse bran, and (3) fine bran after endogenous enzymes activation followed by Osborne fractionation, (4) MW marker.

Characterization of extracted proteins collected from the Osborne fractionation supernatants (section Co-extraction of Starch and Proteins by Osborne Fractionation) and enzymes activation followed by Osborne fractionation (section Combination of Wheat Bran Enzymes Activation and Osborne Fractionation to Increase Proteins Extraction) was performed using SE-HPLC (Supplementary Figures 1–4), SDS-PAGE (Figure 8), and FT-IR (Supplementary Figures 5, 6). The water soluble albumins/globulins have a broad molecular weight (MW) range in which, most of the proteins have reported MWs between 15 and 30 kDa (De Brier et al., 2015), which is in accordance with the peaks at 16, 22, and 28, kDa recoded by SE-HPLC (Supplementary Figure 1). The extracted protein content in the supernatant corresponding to ethanol extracted prolamin was very low, with no peaks visible on SDS_PAGE, and no proteins from the prolamin fraction were identified (Supplementary Figure 2). The alkaline extracted glutelins had MWs lower than 30 kDa, and minor peaks at 14 and 21 kDa were found together with a major peak at 8 kDa (Supplementary Figure 3). This may indicate degradation, as the glutelin fraction previously has been reported to contain protein of intermediate and high MW with little protein with MW lower than 30 kDa (De Brier et al., 2015). The better yields after endogenous enzymes activation followed by Osborne fractionation resulted in clearer SE-HPLC profiles that corroborated the previous data but also resulted in additional peaks in the albumins/globulins profile (Supplementary Figure 4). These results showed that the activity of endogenous enzymes did not result in any further degradation of the native proteins to peptides and amino acids, but that some peptides may be present, especially in the glutelin fraction (The MWs of peptides should be below 10 kDa). The collection of non-degraded proteins was confirmed by SDS-PAGE of the albumin/globulin fraction which showed main protein bands in the range 10–30 kDa (and all proteins below 50 kDa) (Figure 8).

Precipitation of the extracted proteins from the albumins/globulins and from the glutelin fractions was carried out to analyze the respective fraction using FT-IR. All FT-IR spectra (Supplementary Figures 5, 6) showed the same pattern for all samples which in principle confirmed the polymeric nature of the proteins in the respective fraction. Generally, the peptide group of the proteins has nine characteristic bands and among them amide A, amide I, amid II, and amide III are the bands of protein infrared spectrum. The wavenumbers between 3,225 and 3,280 cm^−1^ belong to the N-H stretching vibration of amide A (Krimm and Bandekar, 1986). Amid I was found in the range between 1,600 and 1,700 cm^−1^ due to the stretching vibration of the C = O and C-N groups. The regions of 1,510 and 1,580 cm^−1^ belong to stretching vibration of the C-N and the C-C groups of amid II. The wavenumbers between 1,300 to 1,450 cm^−1^ belong to amid III, which results from a mixture of several coordinate displacements (Venyaminov and Kalnin, 1990).

#### Extraction of Remaining Proteins as Hydrolysate Using High pH and High Temperature

Additional steps, including use of conditions that result in extraction of degraded proteins allow better utilization of the total proteins content, adding a second protein product of lower value. After extracting 50 and 42% of the total proteins in native or denatured full-length forms by Osborne fractionation (section Osborne Fractionation) in fine and coarse wheat brans, respectively, using mild condition, the remaining proteins were extracted as a hydrolysate using harsh condition (120°C, pH 12, 1 h). The results showed the extraction of 83.3 ± 1.9 and 86.5 ± 2.6% of the remaining proteins (which correspond to 43 and 53% of the total proteins) from fine and coarse brans, respectively, in hydrolyzed forms (as peptides and amino acids). Hence, approximately half of the proteins could be obtained in non-degraded form, while an additional 43% (of the fine bran proteins) and 53% (of coarse bran proteins) were obtained as a hydrolysate, resulting in an overall extraction yield of 91.1 ± 1.2 and 91.6 ± 1.6% of the total proteins in fine and coarse brans, respectively.

### Insoluble Fiber Determination of Wheat Bran Before and After the Starch and Proteins Extraction

**Figure 9:**
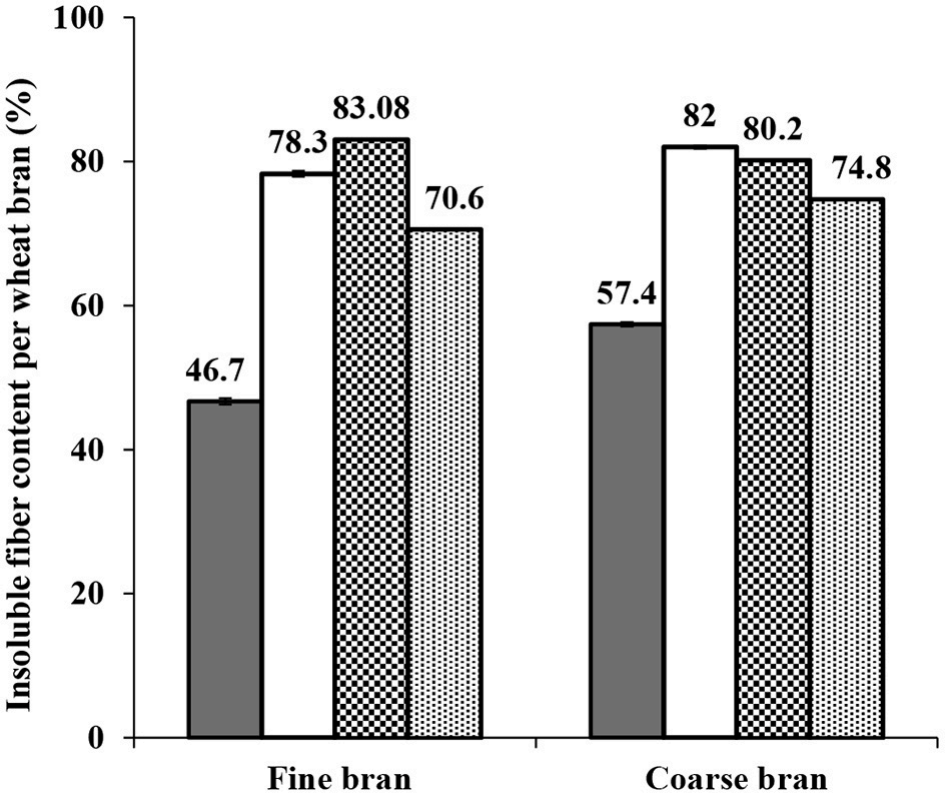
Total insoluble fiber content in fine and coarse brans before and after starch and proteins extractions. Symbols indicate (■) initial content (□) content after destarching process (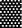) content after Osborne fractionation (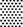) content after proteins extraction at high temperature and pH. The initial content and content after destarching is shown as average from two experiments ± std deviation, while the content after the Osborne fractionation and after the high temperature and pH extraction is based on analysis of single experiments. The Osborne fractionation sequence without endogenous enzyme activation was selected for fiber content analysis. The final fiber content, combining fine and coarse bran can be averaged to 73±3%.

Determination of initial and remaining insoluble fiber was done for each step and fraction. The percentage of insoluble fiber content increased significantly after the de-starching process to 78.3 ± 0.42 and 82 ± 0.2%. A slight further increase was observed after Osborne fractionation (to 83 and 80%), but then decreased during proteins extraction at high temperature and pH (to 70 and 75% in fine and coarse brans, respectively), indicating some solubilization of polysaccharides from the fiber fraction, resulting in co-extraction of insoluble fiber during the final (high temperature and pH) extraction step (Figure 9).

## Discussion

The expected shift from fossil to renewable resources for the production of both chemicals and food, highlights the need of efficient utilization of agricultural resources, including the valorization of underutilized fractions. Wheat bran is to date an underutilized resource. Currently, the main use of bran is as low-value ingredient for human and animal consumption (Prinsen et al., 2014; Pruckler et al., 2014), and can even be burnt to produce energy. This material has, however, potential for production of higher value products. In this paper, we present protocols to increase the use of wheat bran, by introducing sequential protocols allowing the separation of bran components into fractions rich in starch, lipids, proteins and fibers.

Extraction of starch using water as solvent was an easily applied step at low temperature that allowed separation of insoluble starch via filtration. This methodology has previously been described by Du et al. (2009). At 30°C for 30 min a more than 70% yield of extracted starch was obtained, which was in agreement with the starch extraction yield from coarse bran in this study. Extraction of starch by swelling the brans in water at 55°C for 4 h as a first step, followed by enzymatic hydrolysis steps has been described by Merali et al. (2015). Extraction using these conditions resulted in high yield of extracted starch (82%). However, the 55°C temperature might cause gelatinization of the starch during the extraction process, and as observed in our study the higher solubilization in the water phase made the starch more difficult to recover. Even higher yields of extracted starch (90%) from wheat bran have been reported using a combination of wet-milling and extraction with 70% ethanol combined with H_2_O- toluene. However, this solvent combination is not desirable from an environmental perspective (Xie et al., 2008). The mild conditions in terms of solvent, temperature and pH, allowed separation of the starch from the main part of proteins and fibers in the bran, for which higher pH and temperature were needed. Since the extracted starch was mainly insoluble in the water fraction used for its separation, it was easily separated by filtration and could be used for different applications. Moreover, starch from wheat bran has been reported to have unique properties (lower gelatinization temperature and lower retrogradation rate) compared to the wheat endosperm starch (Xie et al., 2008), which makes the wheat-bran starch interesting for food industry.

Extraction of lipids was done using SC-CO_2_ extraction which is a mild technique. In general, the oxidation and hydrolysis of lipids and fats cause undesirable odors and flavors. This also shows the importance of finding a mild methodology to avoid degradation of the lipid fraction in the process. The yield of extracted lipids by SC-CO_2_ was almost half of the lipids extracted using a Soxhlet system, in which the extraction yield was almost 100%. However, the Soxhlet extraction is a laboratory method in which the use of hexane as a solvent has beem reported as a drawback (due to hexane being less beneficial to humans and environment, compared to SC-CO_2_; de Castro and Ayuso, 2000). In this study Soxhlet extraction using hexane was used as reference method. One suggestion can be use of hot ethanol (96%) instead of hexane as an alternative for industrial application (Gopalasatheekumar, 2018). The oily extract from wheat bran contains linoleic acid up to 60%. Linoleic acid is polyunsaturated fatty acid, which has a positive action against coronary heart disease (Farvid et al., 2014), suggesting a potential for industrial production of wheat bran oil due to its high content of linoleic acid. Therefore, SC-CO_2_ as an industrial compatible method can be chosen and used.

The protein extraction was the most challenging part, in need of further optimization to be industrially feasible. Modification and optimization of protein extraction methods based on the wheat bran particle size, pH, temperature, sample to solvent ratio, and wheat bran enzymes activation were performed which can be ways to address the challenge to extract native proteins. In addition, the de-starching step may be combined with the protein extraction step, since starch is insoluble while protein is soluble in water and the two components can be easily separated from each other. The yield from extraction of non-degraded proteins by alkali in our study, was in agreement with the extraction yield of 37% obtained by De Brier (De Brier et al., 2015) on roller milled bran, using 0.05 M NaOH at 20°C and the trends were in agreement with the results on ball milled bran described by De Brier et al. (2015), where higher extraction yields were obtained using the finer milled material. The highest yield of extracted total proteins from fine (50%) and coarse bran (42%) by Osborne fractionation in this study was lower than what De Brier and others described (60, 68, and 77% proteins extraction yield from wheat bran with the particle size of 800, 400, and 175 μm, respectively). This is likely due to use of a different milling process. The fine bran in our study was prepared by hammer milling and was a mixture of different particle sizes, < 1 mm.

The impact of the bioprocessing of wheat bran on protein solubility has been described by Arte et al. where activation of wheat bran endogenous enzymes was evaluated at pH 4.5 and 30°C for 24 h followed by washing for 1 h with Tris-HCl 50 mM, pH 8.8 (Arte et al., 2015). In this work, the combination of enzyme activation and Osborne fractionation resulted in an increase in the extraction yield of non-degraded proteins compared to using only Osborne fractionation, but was only effective on bran with the smaller particle size.

A summary of the starch, total lipids, proteins and fiber contents of fine and coarse brans before and after each extraction is given in Table 1. A problem in the processing cascade was the separation of proteins and fiber fractions, for which the majority of the extraction procedures are relatively similar, in terms of e.g., being performed in alkali solution. However, we think that this may be tuned by using shorter time and lower pH to promote protein extraction, leaving the fiber as a remaining insoluble product.

**Table 1:** The starch, total lipids, proteins, and fiber contents of fine and coarse brans before and after starch and protein extraction.

	**Total starch**	**Total protein**	**Total insoluble fiber**	**Total lipids**
**CONTENTS IN FINE WHEAT BRAN (G/100 G BRAN)**				
Initial contents	17.23	14.29	46.7	5.14
After starch extraction at 55°C	5.5	12.92	78.3	4.02
After Osborne fractionation	0	7.13	83.1	9.28
After extraction at high temp and high pH	0	1.05	70.6	11
**CONTENTS IN COARSE WHEAT BRAN (G/ 100 G BRAN)**				
Initial contents	9.75	14.14	57.4	3.64
After starch extraction at 55°C	1.78	12.27	82	2.5
After Osborne fractionation	0	8.21	80.2	8.88
After extraction at high temp and high pH	0	0.98	74.8	10.4

## Conclusion

Different methods for extraction of starch, lipids, proteins, and fiber have been described in scientific literature. However, most of them have focused on selective isolation of single components. A combined approach is slightly more complex, as similar types of methodologies are frequently used to obtain the respective component, while decomposing remaining components. The methodologies in our study were combined in a suggested protocol and performed, both as an individual fractionation, but also based on the scheme suggested to provide a fractionation series allowing sequential retrieval of the fractions of interest. For this purpose, use of mild non-destructive extractions is important to allow retrieval of more than one component from the material.

## Data Availability Statement

All datasets generated for this study are included in the article/Supplementary Material.

## Author Contributions

RS did the majority of the experimental work, and wrote the draft of the manuscript. SS did the lipid extraction experiments together with HA. MG, ML, CT, and EN were participating in the discussions and analysis of the data together with RS, and were also involved in the writing and development of the finished manuscript.

### Conflict of Interest

The authors declare that the research was conducted in the absence of any commercial or financial relationships that could be construed as a potential conflict of interest.
