# Supplementary material for: Evaluation of Sequential Processing for the Extraction of Starch, Lipids, and Proteins From Wheat Bran
Source: Front Bioeng Biotechnol. 2019 Dec 13;7:413. doi: 10.3389/fbioe.2019.00413 (PMC6923635; doi:10.3389/fbioe.2019.00413)
Supplement: Supplementary file 1 [file Data_Sheet_1.docx]

**SUPPLEMENTARY MATERIAL**

**Supplementary Table 1:** Fatty acid methyl ester composition (% distribution) in extracts from different extraction techniques and sample size.

| Fatty acid methyl ester | Soxhlet  extraction | | SC-CO2 extraction | | | |
| --- | --- | --- | --- | --- | --- | --- |
|  |  |  | 150 bars | | 350 bars | |
|  | fine | coarse | fine | coarse | fine | coarse |
| C 16 (P) | 16.73 | 15.09 | 16.22 | 17.22 | 17.11 | 15.75 |
| C 18 | 0.63 | 0.72 | 1.19 | 0.67 | 0.66 | 0.75 |
| C 18:1 (O) | 12.42 | 15.21 | 17.84 | 13.05 | 12.88 | 15.35 |
| C 18:2 (L) | 59.70 | 60.08 | 57.87 | 60.31 | 59.03 | 59.61 |
| C 18:3 (α-L) | 6.03 | 4.97 | 4.76 | 6.07 | 5.95 | 5.22 |
| C 20:1 | 0.79 | 0.63 | 0.50 | 0.83 | 0.81 | 0.63 |
| Others | 2.46 | 2.40 | 0.77 | 0.60 | 2.39 | 1.83 |

**Supplementary Figure 1:** Molecular weight distribution of extracted albumin/globulin fraction from (A) coarse bran, (B) fine bran, and (C) as mobile phase by SE-HPLC.

**Supplementary Figure 2:** Molecular weight distribution of extracted prolamin fraction from (A) coarse bran, (B) fine bran, and (C) as mobile phase by SE-HPLC.

**Supplementary Figure 3:** Molecular weight distribution of extracted glutelin fraction from (A) coarse bran, (B) fine bran, and (C) as mobile phase by SE-HPLC.

**Supplementary Figure 4:** Molecular weight distribution of extracted (A) albumin/globulin and (B) glutelin fractions from fine bran after enzyme activation and Osborne fractionation by SE-HPLC.


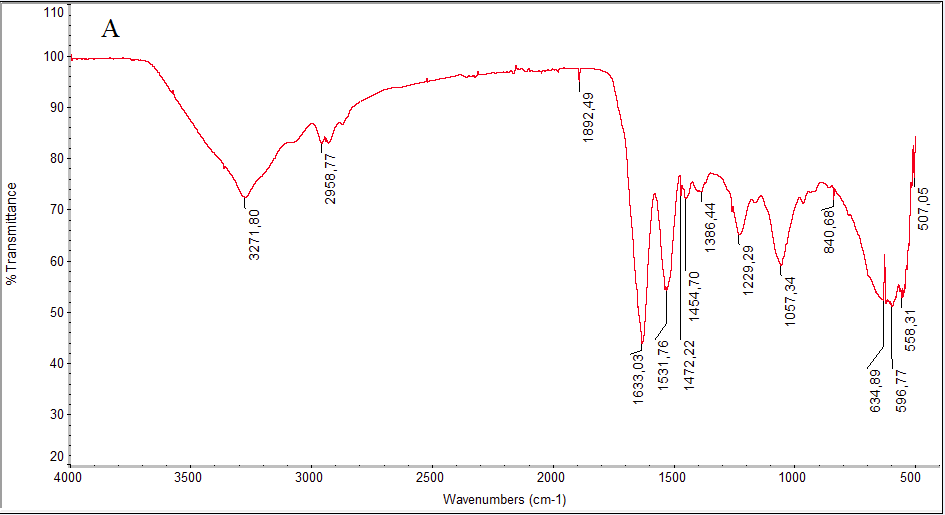


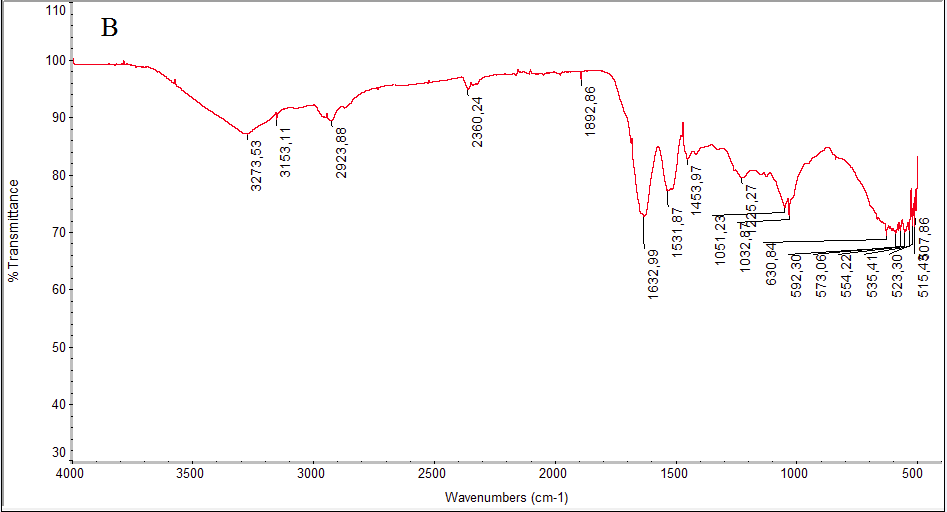


**Supplementary Figure 5:** FT-IR spectra of extracted (A) albumin/globulin, and (B) glutelin fractions from coarse bran after precipitation.


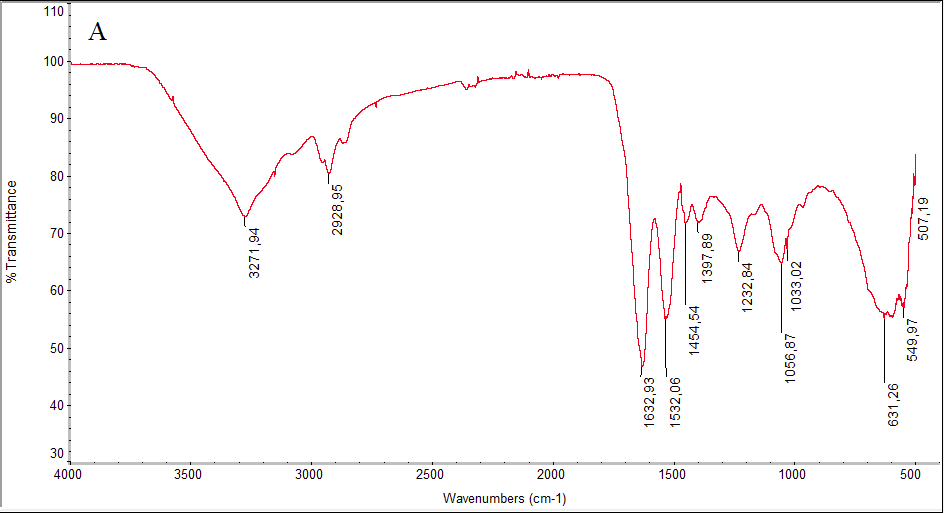


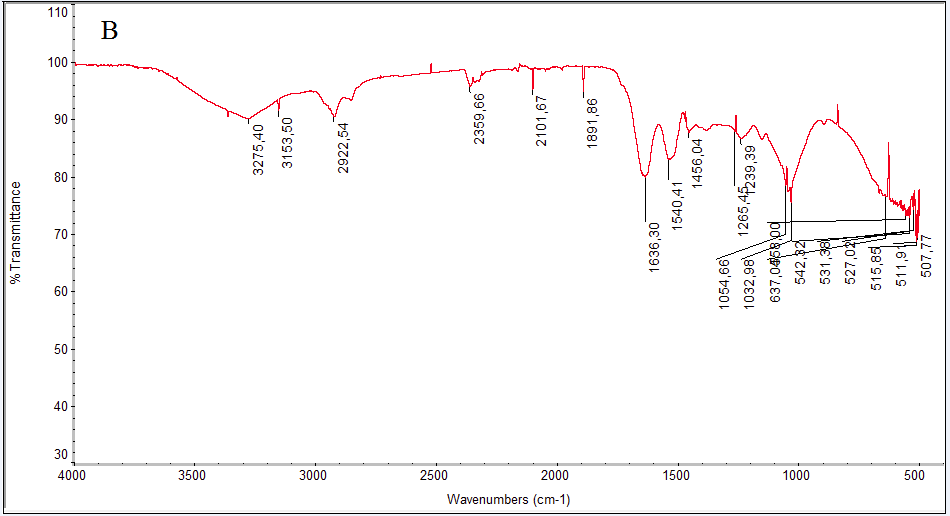


**Supplementary Figure 6:** FT-IR spectra of extracted (A) albumin/globulin, and (B) glutelin fractions from fine bran after precipitation
